# Editorial: Bone metastases in endocrine cancers: advances in diagnosis, treatment, and prevention

**DOI:** 10.3389/fendo.2026.1953774

**Published:** 2026-08-28

**Authors:** Chandi C. Mandal, Julie A. Rhoades

**Affiliations:** 1 Department of Biochemistry, School of Life Sciences, Central University of Rajasthan, Ajmer, India; 2 Tennessee Valley Healthcare System, Department of Veterans Affairs, Nashville, TN, United States; 3 Department of Medicine, Vanderbilt University Medical Center, Nashville, TN, United States

**Keywords:** advanced imaging, biochemical/molecular markers, bone metastases (BM), endocrine cancer, skeletal complications

## Introduction

Clinical assessment, accurate detection, and management of endocrine cancer-induced bone metastases (BM) and skeletal-related events (SRE) largely relate to the patient outcomes and quality of life. Dysregulated bone metabolism leads to changing bone microarchitecture and strength that enhance bone fragility (1, 2). In addition, disseminated tumor cells and therapies can alter intercellular communication between cells in the bone microenvironment, leading to an imbalance in bone homeostasis. BM cells promote either osteoclast activity or abnormal osteoblast function or both to develop osteolytic and/or osteoblastic metastatic diseases (3, 4). Recent advancements in diagnosis/detection, treatment modality, and surgical techniques display significantly improved prognoses for cancer patients with metastatic bone disease. This editorial topic underlined that the combination of advanced imaging and biochemical and molecular diagnostic/prognostic factors is the need of the hour to provide a precise clinical assessment and subsequent person-specific treatment for better efficacy.

## Relationship between PPGL bone metastases and SDHB gene

Similar to breast, prostate, and other endocrine cancers, bone metastasis is a frequent event in pheochromocytoma and paraganglioma (PPGL) cancer patients. Li et al., study reported that 75% of these patients develop BM with multiple lesions with a high frequency in the spine, and create skeletal complications, including pain. The SDHB gene mutation was found to be an independent risk factor for bone metastasis of PPGL cancer patients. Moreover, Contreras-Saldarriaga et al., research team further supported that the genetic mutation of the SDHB gene links with the diagnosis and accurate prediction of BM for PPGLs.

## FGF23, hypophosphatemia and soft tissue tumor-induced osteomalacia

Diaz-Garzon Marco et al., described that excessive phosphate excretion results in hypophosphatemia, low vitamin D reabsorption, and elevation of ALP, which in turn develops osteomalacia. In X-linked hypophosphatemia, mutation of the PHEX gene elevates FGF23 expression, which in turn promotes excessive renal phosphate wasting, leading to developing skeletal disorders including osteomalacia. Similarly, soft tissue mesenchymal tumors also elevate FGF23 to create tumor-induced osteomalacia. In both cases, serum phosphate, phosphaturia, FGF23, ALP, PTH, vitamin D, serum calcium, and calcium-excreted rate calcemia are linked with better diagnosis and management of osteomalacia. FGF23, fragmented FGF23, bone-specific ALP, and patient age are to be crucially considered for interpreting clinical findings. Tao et al., study found 33 cases of soft-tissue tumor-induced osteomalacia (TIO) of the trunk, with a mean age of 49.7 years and a high prevalence in males. A case study confirmed release from bone pain and returned blood phosphorus level to normal upon removal of the phosphaturic mesenchymal tumor. Immunohistochemistry showed that spindle cells were positive for vimentin, CD56, Ki-67, FGF23, SSTR2A, and SATB2, but negative for CD34. However, careful physical examination and advanced MRI, CT, SPECT/CT imaging are required to detect soft tissue tumors. For instance, ^68^Ga-DOTATATE PET/CT successfully detected tumors in 24% of patients with TIO. For lesions, ^68^Ga-DOTATATE PET/CT is mostly beneficial with added sensitivity compared to the octreoscan SPECT/CT and 18FDG-PET/CT. Moreover, anti-FGF23 antibody treatment may improve the quality of life.

## Diagnosis of bone metastases of endocrine and small intestine neuroendocrine cancers

Early diagnosis and precise detection of BM are still challenging. Huang et al., suggested that the combination of advanced imaging and biochemical/molecular parameters provides a precise clinical evaluation and recommends specific treatment for an individual for better efficacy. Sensitivity and correctness in BM detection largely vary in endocrine cancer types and also in imaging types like CT, MRI, PET, ^68^Ga-DOTATATE PET/CT, Ga-PSMAPET/CT. For example, PSMAPET/CT showed 90% sensitivity for prostate cancer, but was not for thyroid and neuroendocrine tumor-associated BM, whereas ^68^Ga-DOTATATE PET/CT worked better for G1/G2 neuroendocrine tumor induced BM, but was not suitable for breast and prostate cancer. However, bone-modifying agent bisphosphonates and denosumab are used as therapy for bone metastasis of various endocrine cancers.

Mathew et al., pointed out that the prevalence and clinical features of small intestinal neuroendocrine tumor (siNET) BM are not well studied. These BMs significantly affect SRE and overall survival (OS) of the patients with siNETs. This study reported 34% BM of cancer patients with stage IV. Functioning tumors are more frequent in BM patients. OS was lower in patients with BM, independent of age, sex, or tumor grade. However, SRE (12%) was found in osteolytic BM. Anti-resorptive therapy may reduce SRE and improve OS.

## Thyroid cancer bone metastases: meningeal metastatic tumor and meningioma

Liu et al., study revealed that endocrine epithelial follicular thyroid cancer (TC) rarely develops a meningeal metastatic tumor with cranial bone destruction, and it is often misdiagnosed as meningioma. Surgical removal achieved favorable initial disease control with a chance of recurrence. Yao et al., documented that TC patients with BM significantly affect cancer specific survival (CSS) and SRE. The study of 82 patients with TC and BM exhibited worse CSS with tumor size, age, and extraosseous metastases. Systemic integrative therapy could be a better choice for managing BM and SRE.

## Consideration of radiation osteitis and osteonecrosis for bone disease treatment

Chemotherapy, monoclonal antibody, and radiation therapy are the best treatment options for advanced malignancy. Xue et al., noticed a presence of radiation osteitis (RO) which shows SRE, which may be misrepresented as bone metastasis in case of advanced colorectal cancer. Imaging features of RO must be carefully scrutinized to differentiate it from BM. A few features of RO are osteocyte destruction, lack of osteoblasts, and new osteoid formation, where the CT scan finds decreased bone density and patchy sclerosis. In case of RO, no markers have been addressed yet, but diagnosis of BM usually includes ALP, N-terminal propeptide of procollagen type I, C-terminal propeptide of procollagen type I, tartrate-resistant acid phosphatase, bone sialoprotein, and so on. However, in the case of RO, ALP was normal during the treatment period. The RANKL monoclonal antibody denosumab is a frequently prescribed drug to prevent bone thinning, especially for osteoporosis and cancer related skeletal resorption. Denosumab is used alone or in combination with other bone-modifying agents, including bisphosphonates and glucocorticoids. However, Bai et al., documented that the higher dose (120 mg denosumab) develops more osteonecrosis of the jaw compared to the lower dose (60 mg). This osteonecrosis is more prevalent when denosumab is used with other anti-resorptive therapies like bisphosphonates.

## LRG4 and inflammatory markers for bone diseases

G protein-coupled receptor protein LRG4 plays an important role in bone remodeling. Huang et al., deliberated that the deficiency of LRG4 accelerates osteoclast activity, leading to osteoporosis. Similarly, knockdown of LRG4 showed delayed bone formation. LRG4 expression was found to be elevated in various cancers, including breast, prostate, lung, and multiple myeloma, and it promotes epithelial to mesenchymal transition (EMT) to favor cancer metastasis. This double-edged sword positively regulates bone homeostasis but enhances tumor potential and its metastasis. It participates in regulating many crucial signalling pathways, including RANKL-RANK-NFATc1, GSKβ-Wnt, TGFβ/BMP signaling in bone resident osteoblasts and osteoclasts, and in cancer cells. Thus, targeting LRG4 might be a promising strategy, especially for cancer induced bone metastasis. Chen et al., suggested that the clinical parameters like serum PSA and Gleason score may not truly predict and/or correlate with the degree of severity of bone metastasis of prostate cancer. Thus, additional markers are required to establish the accurate prediction and/or therapy response. A few inflammatory markers, including TPSA, FIB, HB and HAR along with Gleason grade, are assigned as independent risk factors for bone metastasis. A comprehensive model study suggested a novel nomogram where the combination of both inflammatory and clinical markers predicts better for prostate cancer bone metastasis.

Certainly, this noteworthy Research Topic updates the current scenario of advancements in diagnosis and treatment of BM and skeletal complications induced by endocrine cancers, and argues that the combination of advanced imaging and biochemical/molecular markers certainly allows for the precise identification of the clinical onset of the cancer-associated bone diseases.
